# Endosymbiosis before eukaryotes: mitochondrial establishment in protoeukaryotes

**DOI:** 10.1007/s00018-020-03462-6

**Published:** 2020-02-01

**Authors:** István Zachar, Gergely Boza

**Affiliations:** 1 Evolutionary Systems Research Group, Institute of Evolution, Centre for Ecological Research, Klebelsberg Kunó str. 3., Tihany, 8237 Hungary; 2 MTA-ELTE Theoretical Biology and Evolutionary Ecology Research Group, Department of Plant Taxonomy and Ecology, Eötvös Loránd University, Pázmány Péter sétány 1/c, Budapest, 1117 Hungary; 3 Center for the Conceptual Foundations of Science, Parmenides Foundation, Kirchplatz 1, 82049 Munich, Germany; 4 Evolution and Ecology Program, International Institute for Applied Systems Analysis (IIASA), Schlossplatz 1, 2361 Laxenburg, Austria

**Keywords:** Mutualism, Endosymbiosis, Prokaryotes, Mitochondria, Eukaryogenesis

## Abstract

Endosymbiosis and organellogenesis are virtually unknown among prokaryotes. The single presumed example is the endosymbiogenetic origin of mitochondria, which is hidden behind the event horizon of the last eukaryotic common ancestor. While eukaryotes are monophyletic, it is unlikely that during billions of years, there were no other prokaryote–prokaryote endosymbioses as symbiosis is extremely common among prokaryotes, e.g., in biofilms. Therefore, it is even more precarious to draw conclusions about potentially existing (or once existing) prokaryotic endosymbioses based on a single example. It is yet unknown if the bacterial endosymbiont was captured by a prokaryote or by a (proto-)eukaryote, and if the process of internalization was parasitic infection, slow engulfment, or phagocytosis. In this review, we accordingly explore multiple mechanisms and processes that could drive the evolution of unicellular microbial symbioses with a special attention to prokaryote–prokaryote interactions and to the mitochondrion, possibly the single prokaryotic endosymbiosis that turned out to be a major evolutionary transition. We investigate the ecology and evolutionary stability of inter-species microbial interactions based on dependence, physical proximity, cost–benefit budget, and the types of benefits, investments, and controls. We identify challenges that had to be conquered for the mitochondrial host to establish a stable eukaryotic lineage. Any assumption about the initial interaction of the mitochondrial ancestor and its contemporary host based solely on their modern relationship is rather perilous. As a result, we warn against assuming an initial mutually beneficial interaction based on modern mitochondria–host cooperation. This assumption is twice fallacious: (*i*) endosymbioses are known to evolve from exploitative interactions and (*ii*) cooperativity does not necessarily lead to stable mutualism. We point out that the lack of evidence so far on the evolution of endosymbiosis from mutual syntrophy supports the idea that mitochondria emerged from an exploitative (parasitic or phagotrophic) interaction rather than from syntrophy.

## Introduction

While the idea of endosymbiotic origin of organelles has emerged early in the twentieth century [1-3], it was a little more than 50 years ago that Lynn Margulis [4] has established the prokaryotic ancestry of mitochondria and plastids and the endosymbiotic origin of eukaryotes. According to theories, a bacterial species merged with another host microbe, presumably an archaeon, giving rise to eukaryotes. Eukaryotes are ancestrally nucleated, mitochondriate and seems to be phagocytotic since before the last eukaryotic common ancestor (LECA; [5-7]); however, their order of acquisition is unknown. Whether mitochondria were prerequisites or results of eukaryogenesis is a matter of ongoing debate, aggravated by missing crucial details [8, 9]. They, nevertheless, have a fundamental role in the functioning of most eukaryotic cells and they might had a pivotal role in the origin and early evolution of eukaryotes. Arguably, during mitochondrial integration, a new organism and a new level of complexity have emerged from individually reproducing cells, qualifying it as a major evolutionary transition [10, 11].

Consequently, mitochondria became the flagship example of endosymbiosis between prokaryotic partners, even though the (presumably) singular origin of eukaryotes constitutes a single example. Other endosymbioses, involving only prokaryotes living outside of eukaryotic cells, are unknown thus far (except for a scarcely documented case in Cyanobacteria, see Table 1). This suggests that the origin was either a genuinely singular event or alternative lineages have disappeared. It is intriguing why endosymbioses of prokaryotic partners (presumably evolving many times during the ~ 3.5 billion years) have never reached a success comparable to eukaryotes and why current eukaryotic diversity traces back to a single origin. As a result, it is precarious to draw general conclusions about prokaryotic endosymbiosis based on this singular example. Here, we explore prokaryotic endosymbioses starting from general mechanisms assumed to apply to any endosymbioses and discussing special properties relevant to unicellular partners.

Interspecific reciprocal beneficial interactions, in which both parties receive net benefit are quite common and are referred to as mutualism [12], while cooperation is more commonly used for intraspecific interactions. While definitions vary, symbiosis is often described as a prolonged, intimate relationship of different species in which partners live in physical contact, often physiologically integrated (in most or all of their life cycles) [12] and have already started to adapt to each other evolutionarily. While mutualism refers to the cost–benefit balance of the interaction, symbiosis refers to physical contact and dependence. Consequently, symbiosis is not necessarily mutually beneficial and can be asymmetrical with a conflict of interest (e.g., parasitism) or without a conflict of interest (e.g., commensalism). Endosymbiosis covers a spectrum of partially or fully obligate interactions with various levels of dependence, of which the absolute extremum is a mutually obligate, physically integrated partnership with an exclusively vertically transferred symbiont [13]. Endosymbiosis can also range from being mutually beneficial to exploitative. For that matter, one can argue that modern eukaryotes simply exploit mitochondria by tapping into their ATP reserves (see [14]). These definitions apply regardless of parties being unicellular or multicellular.

For an endosymbiotic partnership to be evolutionarily stable, there must be a selective advantage, so that the pair is favored by selection over individually reproducing parties; otherwise, the former could be outcompeted by the latter. However, endosymbiotic partners (e.g., ancestral mitochondria and host) are not related genetically, and thus, they do not share genes by common descent and one cannot expect predisposition towards cooperation. For stable endosymbiosis, cells of different lineages must align their interests and evolve synergies based on their different properties. In transitions theory, such partnerships are called egalitarian [15].

Different genetic backgrounds generally translate into different metabolisms. Such differences can enable division of labor if there is metabolic complementation, with minimized conflict and pre-aligned reproductive interests [13]. Metabolic exchange due to complementation (e.g., syntrophy), is assumed to be a key factor in the establishment of microbial consortia, biofilms, and mats [16]. It is widely assumed that syntrophic interactions may have contributed enormously to the emergence of major endosymbiotic transitions including the origin of eukaryotes. In particular, various hypotheses of eukaryogenesis assume ancient syntrophic interactions [17-19]. In syntrophy, organisms jointly catabolize a substrate that neither party can catabolize alone [20] (for a review on classification, see [21]).

We have recently reviewed our present knowledge and its gaps on the origin of mitochondria and eukaryotes [8]. Here, we explore the forms of interactions leading to symbiosis of unicellular (prokaryotic or eukaryotic) partners and general mechanisms that facilitate and stabilize such interactions, with a focus on potential endosymbiosis, especially of mitochondrial origins. Given that initial ecological interactions and conditions are debated, we extend our scope to a wide range of mechanisms that could set off such a partnership. We do not reiterate but call attention to the many unknowns of the nature and relation of ancestral symbiont and host; for further details, we refer the Reader to reviews [22-24].

Establishing and maintaining an interaction, internalizing a partner, and driving the partnership to mutual benefits entail different issues and require different solutions. We review these issues and the mechanism that may solve them, especially in case of the origin of mitochondria. First, we give an account of prokaryotic endosymbioses and briefly discuss the various benefits that can power symbiotic interactions. Then, we review mechanisms that can help strong pairwise associations to emerge and stabilize. We briefly discuss central control mechanisms that cause irreversible dependency, ultimately leading to organellogenesis. Next, we provide a detailed investigation of certain issues pertaining prokaryotic endosymbioses, discussing their relevance to various hypotheses of the origin of mitochondria. We conclude the paper pointing out the major issues of origin hypotheses and how researchers should employ evolutionary thinking and experimentation in figuring out details of prokaryotic endosymbiosis and organellogenesis.

### Prokaryotic endosymbioses

While endosymbiosis is widespread among eukaryotes and between eukaryotes and prokaryotes [25, 26], endosymbiosis among prokaryotes is rare [25, 27, 28]. In contrast, there seems to be a diversity of obligate ectosymbiotic organisms belonging to both archaea and bacteria that depend on a prokaryotic partner [29-32]. However, no contemporary prokaryotic endosymbionts to archaeal hosts are known [33] and the known endosymbioses among bacteria [25] are not comparable to mitochondria. These are either limited to ectosymbiotic or periplasmic contact or embedded in a eukaryotic “overhost” [34], where the immediate host is reduced so much that the inner symbiont is more the symbiont of the overhost. It is debatable how strong an analogy these instances represent of exclusively prokaryotic endosymbioses. The few prokaryotic examples are listed in Table 1, indicative of how little we know of archaeal symbioses yet.

**Table 1:**
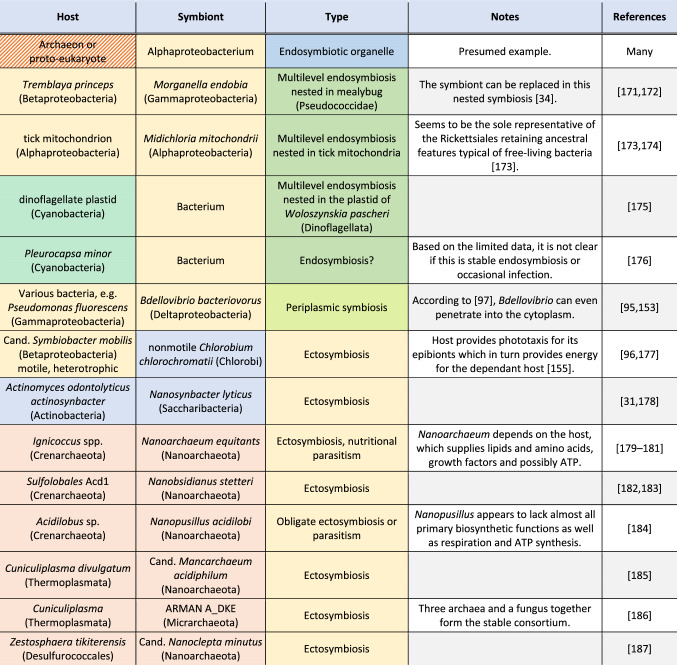
List of known (endo)symbioses of prokaryotic hosts and symbionts that evolved (presumably) after mitochondria

For reference, we have included the mitochondrial origin as well. Note, that in most cases, the symbiosis is epibiotic and true endosymbiosis is mostly restricted to prokaryotic hosts embedded in eukaryotic cells. Yellow background of Host and Symbiont columns stands for Proteobacteria, green for Cyanobacteria, blue for other bacteria, and red for Archaea, striped for unknown. Blue background for Type column stands for endosymbiotic organelle, green for endosymbiosis, and yellow for ectosymbiosis

If we assume that the host was not a proto-eukaryote, the only (putative) example remaining of a stable endosymbiosis of prokaryotes is the eukaryotic cell with its mitochondria. And the only cases of eukaryote–prokaryote partnerships that led to new organelles are the two primary plastids, those of Archaeplastida and *Paulinella* [35]. Unfortunately, we lack intermediates of the history from first to last eukaryotic common ancestor [9]. The limited number of prokaryotic examples might give the false impression that mitochondrial integration is the stereotypical form of endosymbiosis and its mutually beneficial state of affairs stems directly from ancestral mutualism. As a consequence, the eukaryogenetic endosymbiosis is often assumed to be of mutualistic nature. Symbiogenetic theories generally assume an initial syntrophic interaction between a prokaryotic (often archaeal) host and a bacterial partner (e.g., the hydrogen hypothesis [17], the syntrophy hypothesis [18], the reverse flow model [19], and others [36-40]). However, assuming an initial mutually beneficial interaction has at least three issues.

**Fig. 1:**
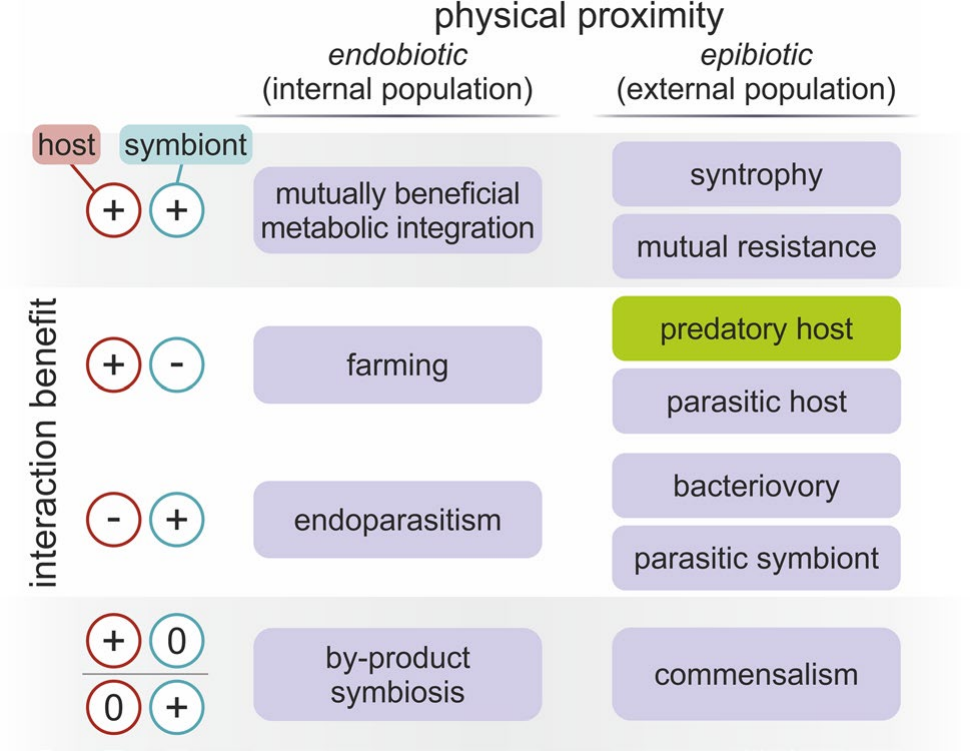
Prototypical ecological interactions of host and symbiont enabling endosymbiosis, along two orthogonal dimensions: symbiosis (physical contact) and net benefit of the interaction of populations (mutualism). All endobiotic partnerships are technically endosymbiosis. It is trivial that endosymbiosis requires a transition from ecto- to endobiosis, but it is not necessary that the interaction converges to (+|+) . Transition between states is always continuous, rather than stepwise, and boundary states are not separating (i.e., one can go from syntrophy to by-product symbiosis). Mutually beneficial syntrophy, if internalized, could naturally yield metabolic endosymbiosis. Similarly, farming becomes mutually beneficial when the farmed partner survives in an otherwise lethal environment thanks to the host. On the other hand, two steps are needed to turn phagocytosis into mutually beneficial endosymbiosis: first, a mechanism is needed to turn the host to farmer then another mechanism must ensure benefit for both parties. Green box indicates where phagocytosis seems indispensable. We exclude mutually disadvantageous cases (−|−) as those are unlikely to lead to association-level advantages

First, there is no direct evidence for the validity of syntrophic scenarios (neither is there for parasitic or phagotrophic scenarios; see [8]). Second, obligately syntrophic partnerships can turn out to be facultative, if the required substrates are provided by another source or partner [41-43]. Third, the path to endosymbiosis cannot be explained by simply characterizing the end state. It is the result of millions of years of coevolution during which properties, costs, and benefits have changed as the association became increasingly intimate and dependent. As the sign of ecological interaction (beneficial or exploitative) and the proximity in which parties live (internally or externally) are orthogonal dimensions, evolution could independently advance along them. Multiple ecological routes can converge to stable, mutually beneficial endosymbioses (see Fig. 1 and [13, 44]). Even in one taxon, there could be multiple independent origins of endosymbiosis (as in trypanosomatids [24] or rickettsiales [45]), indicative of the ease of which some species team up and the variability of the initial conditions permissive toward integration. The variety of mitochondria-related organelles (MROs) and their polyphyletic origins in different eukaryotic clades [46] indicate their capability for quick adaptation to new niches, which effectively mask the ancestral state of all modern mitochondria [47]. We, therefore, stress that the evolutionary history of any endosymbiosis cannot be deduced solely by looking at the existing relationship. This is particularly true to mitochondria which have evolved to the extreme, both in terms of gene-reduction (think of mitosomes) and in terms of efficiency (ATP exchange). The tragedy of mitochondrial origin is that the initial conditions and the order of eukaryotic inventions are all hidden behind the event horizon of LECA [33].

**Fig. 2:**
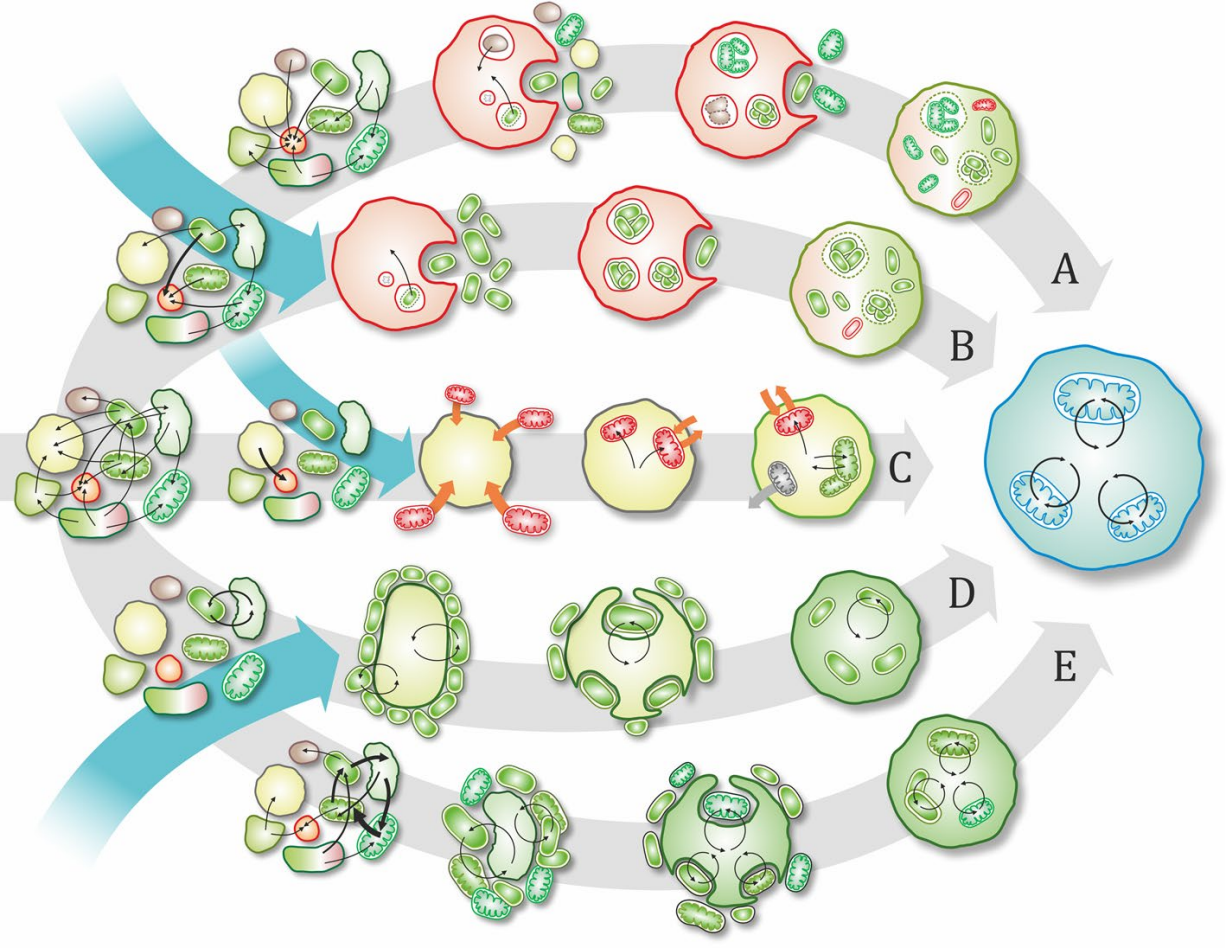
Hypothetical evolutionary scenarios of prokaryotic endosymbiosis. The sign of the partner in the ecological interaction is indicated by the cell color: green is beneficial toward the partner, red is exploitative, and yellow is neutral. The blue cell indicates a fully integrated symbiont species within the host cell, a new evolutionary unit. The interaction either starts in a dense multi-species biofilm where there is a network of various interactions among species (gray arrows) or as a pairwise interaction of two free-living species (blue arrows). A, B: Initial interaction is exploitative as the host feeds on the partner species (this could happen by assuming phagotrophy or external digestion). If the symbiont can maintain its population internally against host culling, there is a chance for coevolution and endosymbiosis. If the original interaction is non-specific, host is expected to maintain a diverse internal population (A). Exploitation can also emerge as a specific pairwise interaction either in the biofilm or of free-living forms (B). Resulting symbionts could defect due to mutations (red symbionts). C: If symbiont is the exploitative partner (a parasite), its entry into the host does not depend on the host's ability to phagocytose. A prolonged interaction could lead to temperated parasite costs, and, ultimately, to a tamed parasite (green symbiont) that stays with the host. D, E: Initial interaction is mutually beneficial, e.g., syntrophy. Partners can be specific and strictly pairwise (D) or work together as a multi-species network to utilize resources (E). In case endosymbiosis emerges from a non-specific network of interactions of multiple species, one expects the resulting integrated pair has greater symbiont, organelle, or genetic diversity (especially in case of nucleated eukaryotes)

For most endosymbioses, it is an open question if partners (extant, prokaryotic or eukaryotic) first established a symbiotic relationship funded on their initial parasitic (+|−) or commensalistic (+|0) interaction, or, alternatively, the initial relationship was already mutually beneficial (+| +) giving way to gradual coevolution to symbiosis [48-50]. Other pathways are also possible [13, 49]. It is reasonable to start investigating any endosymbiosis at the absolute initial stage, when the two species were living independently. Endobiotic interactions, even parasitism, often evolve to associations with mutual benefits [51, 52]. Endobiotic interactions were surely preceded by epibiotic interactions. The practical question is: which came first, “endo” or “symbiosis”? That is, whether a non-cooperative interaction became endobiotic first, after which entirely different mechanisms ensured benefits for both parties or it was an epibiotic association that evolved to mutual benefits and later turned to engulfment (see Fig. 1). Parasitism is generally present among prokaryotes; therefore, integration could happen prior to cooperation. While phagocytosis has thus far only been found in eukaryotes (and traces back to at least LECA [7]), it is not unlikely that the host relied on it to acquire the ancestor of the mitochondrion [53, 54]. Figure 2 illustrates some possible scenarios of mitochondrial origins.

### Benefits of interactions

Biological interactions are complex, forming a continuum from fitness-reducing parasitism to fitness-increasing beneficial symbiosis. However, host–symbiont interactions are not necessarily mutually beneficial; they could be context-dependent [55, 56] with temporal shifts between mutualism and antagonism [55, 57]. For example, photosynthetic symbionts may take more resource than they produce in the dark phase [24, 58]. When the cost of interacting with a partner becomes higher than the benefit obtained from that interaction, mutualism can turn into unilateral exploitation [55]. Therefore, context-independent benefits ensure more robust interactions than environmentally driven, context-dependent ones, although the former may be rare in nature (see [14]). For example, a host is expected to evolve stronger dependence in nutritional mutualism with continuous benefit as opposed to defensive benefits that are beneficial only under certain conditions (Table 2) [59]. Nevertheless, context-dependent benefits can be adaptive if the geometric mean fitness over a longer timespan of environmental fluctuations is larger than those of symbiont-free competitors’. For example, if the overall daily benefit is sufficiently large, hosts farming photosynthetic symbionts have a selective advantage over nonfarming phenotypes [58].

**Table 2:** Benefits based on services in microbial symbioses

Benefit type	Description	Examples
Protection		
Habitat (protection against abiotic factors)	Providing safe environment by removing, neutralizing, or buffering environmental risks and fluctuations including harmful products and agents [114, 188]	Providing extracellular adhesive polymer for biofilm structure [67, 189] Detoxification or sinking of a reverse-inhibiting product *Dictyostelium* carrying and dispersing food bacteria to reintroduce them at a new site to provide new food crop, also helping the symbiont to survive and spread [190] Parasite inducing resistance in the host, e.g., *Holospora*-infected *Paramecium* hosts can tolerate more heat than the uninfected due to symbiont-induced expression of heat‐shock resistant proteins [191]
Resistance (protection against biotic factors)	Restricting or inhibiting access of biological agents, by providing structural or antibiotic resistance	Extracellular matrix of the biofilm [192] Species providing resistance for the whole biofilm consortium [67], against, e.g., bacteriophages [193] Endosymbionts inducing antibiotic production (mostly in Metazoa, e.g., fungus-growing ants [194])
Nutrition benefits		
Trophic interaction	Feeding on the partner, actively reducing its population size. Direct benefit of providing nutrients for the predator; direct control over its population size	Ciliate*-*alga symbioses that lead to internal, living alga-population [195] (also see under farming)
Metabolite exchange	The metabolic product of one party serves as a resource for another. Localization, spatial patterns, and correlated presence of partners are crucial in rendering metabolite exchange efficient [196, 197]	Free diffusion within the medium: nitrogen-cycling network [42, 67, 198] Directed transfer via transport structures (i.e., reducing loss due to leakiness)
Transportation	Providing transportation for the other party to find resources	*Chlorochromatium aggregatum*: the motile central Betaproteobacterium transports its nonmotile photosynthetic epibionts towards light [177], while the epibionts provide energy for the host [155] The slime mould *Dictyostelium* can set aside and distribute with its spores phagocytized *Burkholderia* cells to colonize new habitats and provide food for the new colony [103]
Indirect benefits and costs	Secondary effects that stem from the primary relationship, but manifest only occasionally or are comparably smaller in effect	Metabolic exchange: the metabolite is the primary benefit, while the host also has access to an alternative metabolic pathway within the symbiont for energy harnessing. Metabolically diverse symbioses (e.g., biofilms), provide the benefit of robustness against harsh or fluctuating conditions [199]. The symbiont or the nutrients coerced from it could provide a more stable and balanced diet for the host [121] Aggregating with cooperative partners is indirectly beneficial as it reduces interactions with non-cooperative individuals [200] Predatory or parasitic interactions can provide metabolic complementation or acquired resistance against pathogens, that are only beneficial in particular environments
Cross-feeding	Mutualistic metabolism in which partners depend on metabolic products of each other	Removing the product of the partner can be synergistic as it could turn otherwise endergonic reactions to exergonic for the partner [42]; hence, cross-feeding can be seen as a combination of nutrition and protection (detoxification) benefits The majority of biofilm-forming microorganisms are coupled metabolically due to complementarity and dependency (auxotrophy) [42, 67, 201]
Farming	A combination of nutrition and protection benefits: the host can feed on the stored stack; the symbiont is protected against environmental fluctuations and predators	Typical examples are eukaryotic ciliate*-*alga symbioses [195] Theory has demonstrated the selective advantage of storing living prey internally for poor times, especially if resource-poor times are sufficiently long and/or frequent [53] Extant alga-protist examples: the metabolite of the photosynthetic symbiont is only provided during daylight; otherwise, the partnership is costly for the host [58]
Enabling new niches	The host–symbiont association can extend or even enter a new ecological niche where they enjoy reduced competition	Aerobic mitochondria could have provided the possibly anaerobic (microaerophilic) host an opportunity to venture into aerobic habitats, while its direct competitors could not. This has happened to a purple protist [202] According to a hypothesis, it was heat generation by mitochondria that allowed the hyperthermophile archaeal host to find cold yet unoccupied niches [203]

**Fig. 3:**
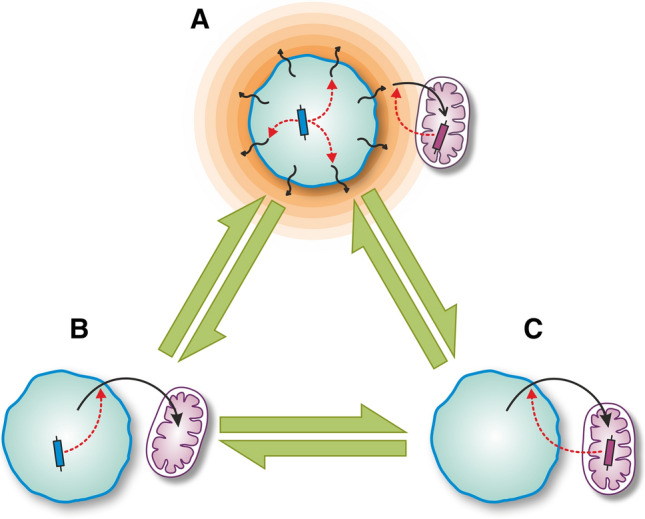
Interaction benefits based on investment. Green arrows indicate evolutionary transitions. A: In by-product benefit, the recipient (purple cell) receives benefit that derives from the by-product of the donor (blue cell). The orange gradient and undulating arrows denote the release and diffusion of the by-product into the environment, which is then picked up by the recipient (black arrow). Both partners have genetic traits (blue and purple rectangles) that can only control their own actions (red arrows). As no costly investment is directed toward the partner [132], no mechanism is necessary to prevent the degradation of the interaction due to exploitative strategies. Since the donor has no interest in the interaction, the benefit for the recipient can only increase in a long-term association by accident or by pseudo-reciprocity (i.e., an investment to enhance by-product benefits [60]), leading ultimately to evolved dependence when the recipient tries to maintain close proximity to harness the by-product more efficiently and stably. In evolved dependence, the removal of the partner from the association causes immediate fitness decrease; however, since one or both of the parties are better off without the other, they can still regain their original fitnesses if separated [62]. B: In invested benefit, the benefit of the recipient (purple cell) derives from the product of the donor (blue cell) which depends on the adaptive trait of the donor (blue rectangle). The maintenance of costly cooperative investments, and ultimately coexistence, becomes an issue: non-investing defectors (cheaters) enjoy a fitness advantage and can outcompete cooperative types [64]. Benefits are expected to be stable or increase if the donor either (i) increases its investment and as a result receives more; or (ii) evolves partner control mechanisms to force adequate or even increasing returns. C: In purloined benefit, the benefit of the recipient (purple cell) depends on its own adaptive trait (purple rectangle) controlling the investment (red arrow). Dependence keeps parties together without quantifiable benefits for the exploited party (called addiction [12]). Different mechanisms may be combined to form pairwise interactions (e.g., A–A yields mutual commensalism, B–B reciprocity, C–B exploitation, and parasitism)

The literature differentiates three classes of benefits in mutualisms based on services: protection, nutrition, and transportation [12] (see Table 2). Interactions can be also categorized based on the source of the benefit and the adaptive trait that controls it: (1) a by-product of an unrelated investment; (2) a direct investment; or (3) purloined [60-63] (see Fig. 3). In invested benefit, the adaptive trait in partner A provides the benefit of partner B. A direct investment is costly for the donor, hence receiving investment without reciprocation can pay off (cheating or free-riding), which leads to social dilemmas [44, 64, 65]. As the trait producing the beneficial by-product for the partner has evolved for purposes other than the mutualistic interaction, by-product benefits are ad hoc and context-dependent, and lack the conflict of interest between parties [60, 66]. In case of purloined (extracted) benefit, both the adaptive trait and the benefit are of one party [61], i.e., it evolves ways to extract benefits from its defenseless partner. The way that eukaryotes exploit mitochondria by employing the adenine nucleotide translocator (ANT) to tap into the symbiont’s ATP pool is an example of purloined benefits. The different benefits, often in combination, have different consequences regarding the stabilization of complex interactions [67, 68].

Competition between partners of different qualities determines the fate of the mutualism: it can result in better services and stronger dependence between partners or it can lead to the degradation of mutualism [57, 69]. To predict the outcome and to understand the underlying dynamics, it is useful to think of mutualism as a bi-directional consumer–resource interaction in which both partners produce resources consumed by the other [65] with two types of a mutualist: high quality and low quality (or exploitative). The mutualistic interaction can be stable if the high-quality mutualist is competitively superior to the low-quality partner. On the other hand, the degradation of the interaction is inevitable if the low-quality partner can outperform the high-quality mutualist [65]. The characteristics of such cooperation-competition trade-offs stipulate the trajectory of coevolution as well as the mechanisms necessary to stabilize these interactions [65]. Various mechanisms were proposed to address these social dilemmas, either punishing the low-quality partner or helping to align the selective interests of the two partners (see partner control mechanisms below).

## Mechanisms facilitating endosymbiosis

Mutualism often originates from asymmetrical, even exploitative interactions [12]; most of them are facultative, and many have relatively recent origins [65]. Obligate mutualisms are rare and considered less stable, since there is a higher chance of (functional) degradation by occasional loss of partner [50, 65, 70]. Symbiosis is shaped by conflicts of interests which are probably harder to manage at the early stage of the association [14, 71]. Consequently, it is unlikely that the ancestors of mitochondria and host first met with perfect metabolic complementarity, so that their symbiosis was immediately mutually beneficial. On the other hand, these specialized obligate symbioses do exist and are persistent for millions of years despite any conflicts, indicative of stabilizing mechanism. In turn, we will discuss mechanisms that can stabilize an emerging though suboptimal interaction so that in can be selected for.

### Group selection in endosymbioses

If groups (associations) form among cells and these groups affect the selection of individual cells, selection appears at multiple levels: individual selection favors the interest of individual cells, while group selection acts in the interest of the associations [11, 72, 73], e.g., of symbiotic pairs. However, multilevel selection almost inevitably leads to between-level conflicts. To better understand group formation, multilevel selection was conceptually characterized into two types, multilevel selection 1 and 2 (MLS1, 2).

In case of MLS1, only temporary groups form that periodically disappear to revert to an unstructured population of cells (also called transient compartmentation) [74]. Facultative (endo- or ecto-) symbioses realize MLS1: partners re-associate better-than-random. Fixed spatial structure of cells can also act as implicit group structure. In dense biofilms, cells are practically immobile and the limited diffusion of exchanged molecules localizes interactions. As a result, mutualist partners stay close and their implicit group can withstand cheating mutants or harmful competitors appearing at group edges [75]. In this case, splitting up the population into explicit, reproductively isolated groups is not required for selection to prefer mutualists [76]. It is yet unknown if endosymbiosis could or have ever evolved in biofilms.

MLS2, on the other hand, involves explicit group structure, i.e., groups that last and reproduce indefinitely. In symbiosis terms, this means exclusive partnership with strict vertical inheritance. If the group is selected for and can stably inherit group-related adaptations, it is a bona fide evolutionary unit (an informational replicator [77]). When obligate codependence of endosymbiotic partners is established, a new unit of evolution emerges [78] and selection of associations dominates over selection of individuals. A major evolutionary transition happens when multilevel selection results in irreversible coupling where individuals forfeit their autonomous replication and gives rise to an association with potential for higher complexity [11]. For group selection to be effective, group members must reproduce together better than random and there must be a selective advantage at the group level. In turn, we will discuss mechanisms that can ensure positive assortativity of partners.

The theory of group selection predicts that the group is favored by selection over individuals if there is a reasonable selective advantage for the group, even if the net of benefits and costs is negative at certain times (i.e., the per capita growth rate of the association is smaller than that of individual cells under certain conditions). Accordingly, the initial partnership does not need to be directly mutually beneficial for both parties at all times, as long as the partnership together enjoys selective advantage averaged over some time or over different environments. Nevertheless, there must be at least a hidden benefit for each party, so that reduced mean fitness in certain periods is compensated. There are at least two general mechanisms to draw such indirect benefits. One is to exploit heterogenous environments, for example temporally fluctuating or spatially differentiated, so that the mean fitness over a wider temporal or spatial range is larger than those of competitors. The other is bet-hedging, that compensates a reduced mean fitness with reduced fitness variance, e.g., with wider tolerance of harsh conditions [79]. This renders the species less prone to extinction in certain selective environments that are truncating, though rare. A prudent strategy counters or even anticipates the effects of a heterogenous environment (see the farming hypothesis, explored theoretically [53]).

### Partner choice mechanisms

**Fig. 4:**
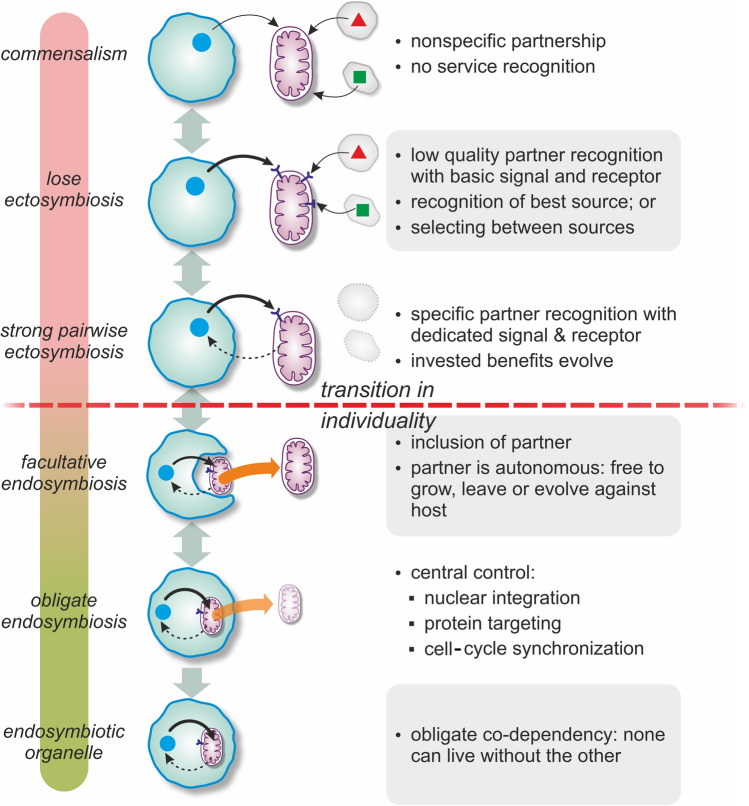
Basic steps of endosymbiosis and organellogenesis. Geometric shapes represent various benefits (e.g., metabolites), solid black arrows represent the source and flow of the various benefits, dashed arrows indicate investments, and colored arrows indicate the option to leave the host. Note that the last step, if involves nuclear integration and protein import, is irreversible

Pre- or post-infection partner choice can stabilize (partially) beneficial interactions [80]. Pre-infection partner choice is based on cues or signals or screening mechanisms to filter partner quality before actually establishing any association with the partner [65, 80, 81]. Quorum sensing, including intra- and inter-species communication, exists both in bacteria and archaea [82, 83]. There is, however, no guarantee that interacting cells are indeed of the cooperative type, as cheating in the form of dishonest signals can arise [84, 85]. Signals can be of two types: diffusive or contact molecules. Surface contact requires close proximity and these signals are usually partner-specific. Diffusive signal molecules can reach a larger number of cells, but are less effective (being diluted easily) and are usually not partner-specific, hence are less reliable. The specificity and reliability required for obligate pairwise symbiosis suggest that surface contact is preferred over diffusive signals (Fig. 4).

Post-infection partner choice is based on conditional investments, and involves various rewarding or sanctioning mechanisms, including the selective termination of the interaction and the possibility of switching partners [65, 86]. The prerequisite of post-infection partner choice is spatial separation of the multiple partners, so that the host can differentiate and then selectively treat high- or low-quality partners; a set-up often referred to as biological markets [13, 80, 87, 88]. The quality of preferable partners depends on multiple factors [65, 87, 88], and often, a low-quality partner is better than no partner at all.

For most of the cases, there is an asymmetry between the mutualist partners in many aspects, such as power of control over the partner, strategic options, availability of alternative partners, etc.[86]. The party with more power or control is expected to gain the higher profit from the interaction, which can even drive the interaction towards unilateral exploitation [58, 65, 88]. Nevertheless, such selectivity by partner control mechanisms can shift the balance in favor of high-quality partners in the population in spite of their competitive inferiority to low-quality partners without the intervention of the mutualist [65]. Additionally, control mechanisms may allow the host to manipulate symbiont behavior and to force higher returns from investing into the symbiont, and may also allow for context-dependent treatment of the partner [56, 58, 89].

### Partner fidelity feedback and internalization

Partner fidelity mechanisms are able to reduce the conflict of interest between partners as the symbiont survival depends on the survival of the host [90]. Increasing investment toward the partner increases the amount or possibility of reciprocated investment, i.e., it is a favor returned [48, 80]. The higher the quality of the mutualist, the higher the chances for survival [65]. Such feedbacks can be interpreted in two time-frames: in-generation or cross-generation. In a generation of a long-term partnership, increasing investments induce higher rates of nutrient flows (in nutrition mutualisms) or higher quality of services (in protection mutualisms) by the partner. Partner fidelity can also manifest as a cross-generational effect, where the investment into a high-quality partner will also benefit the progeny [65].

Cross-generational partner fidelity is usually coupled with vertical (or pseudo-vertical) transmission mechanisms, and is similar in effect to spatial structure: it ensures that offspring can form associations with the same selection of partners as parents did. Strict vertical transmission is very rare (besides endosymbiotic organelles, and some cases of parasitism, like *Wolbachia* in wasps [48, 91]). Imperfect correlation between partners across generations, called pseudo-vertical transmission, is more frequent [48, 92]. Such loose correlations and feedbacks can stabilize mutualism and pave the way for the evolution of perfect cross-generational correlation of partners.

Theory predicts that the evolution of symbiont capture and vertical transmission is driven by host mechanisms to control symbiont transmission [93]. First, because symbiont capture involves the genome reduction of the symbiont while providing increasingly more benefit to hosts, second, because the processes during cell division affecting the distribution and the frequency of reproduction of both parties are controlled by the host, which thus can have the power of selecting which symbionts to transfer (probably restricted to multicellular eukaryotes, e.g., in *Buchnera*–aphid interactions [94].

Undoubtedly, physical inclusion is the most advanced method of vertical transmission, but at the start of a symbiotic partnership, it is rarely available. In most prokaryotic symbioses, physical inclusion never happens, or is limited to a periplasmic space (e.g., *Bdellovibrio* [95], *Chlorochromatium aggregatum* [96]). There are some rare cases where the symbiont can enter the host’s cytoplasm, but, e.g., parasitic *Daptobacter* ultimately kills its host [97]. Phagotrophic eukaryotes could store their captured symbionts in phagosomes (symbiont-bearing vesicles or *symbiosomes* [98, 99]), but whether phagocytosis was the means of mitochondrial inclusion is not known yet. According to some hypotheses, the early host for mitochondria trapped its surface–contact partners in membrane protrusions [100, 101]. In case of a heterotrophic host capable of secreting extracellular digestive enzymes, such entrapment could serve as a poor-man’s phagocytosis [102]. A mixed vertical and horizontal transmission seems to be in effect in *Burkholderia*-infected *Dictyostelium* [103], indicative of facultative endosymbiosis.

### Central control and organellogenesis

As partners become more dependent on each other, and as one party starts to dominate the other, central control evolves. Its ultimate form is the nuclear transfer of symbiont genes, requiring the presence of a nucleus and a mechanism to import proteins from the host cytosol to the symbiont. Evolved dependence on protein and lipid import mechanisms is a sign of endosymbiosis becoming irreversible.

For mitochondrial genes to undergo nuclear transfer, the host must have already been a (proto-)eukaryote. The ancestor of mitochondria could have been acquired before the nucleus, but only with the evolution of the true karyon could compartmentalized, safe transcription (safe from hybridization) be implemented. Symbiont genes relocating into the host nucleus are minimizing the effect of lower level of selection of the multilevel selection situation. With this step, eukaryotes left the prokaryotic domain for good.

After nuclear transfer of genes, it is necessary that proteins not produced by the symbiont anymore find their way back into the symbiont. Usually, this is a translocon-mediated protein import system installed by the host. With a protein import system in effect and a sufficient number of genes transferred to the nucleus, the symbiont could relinquish its protein-coding genes and protein-producing machinery, leveraging its genome. Moreover, this allows the host to introduce proteins of its own interest into the symbiont’s membrane.

The adenine nucleotide translocase (ANT) was probably introduced by the host into the mitochondrial membrane to exchange host-cytosolic ADP with symbiont ATP [36, 104]. ANT was most likely evolved within eukaryotes after the engulfment of the ancestral symbiont [105-107]. It was certainly in the host’s best interest to exploit the symbiont. If, however, it was the symbiont who invented ANT to give up ATP for the host, then any cheater bacterium capable of turning off its ANT while inside the host would have been under positive selection leading to the overpopulation of defecting symbionts, as was pointed out [108]. Group selection could have stabilized against cheaters, but only if a population of endosymbionts payed enough ATP to the host, so that host replicated faster (compared to other host cells); since the symbiont replicated with the host, the benefit was shared [109]. Other partner control mechanisms screening out cheaters are unknown yet.

No prokaryotic analogies of karyogenesis were found yet, though nuclear transfer is known in many eukaryotes [110]. Further features thought to be exclusive to mitochondrial and plastid integration have been recognized in more recent endosymbioses [71, 111, 112], drawing a picture of a continuum from symbiosis to organellogenesis (see Fig. 4). While nuclear integration renders the partnership obligate and irreversible, preventing the escape of the reduced partner, such mechanisms by no means represent an end state. They do not even ensure the survival of the symbiont, as amitochondriate eukaryotes attest. In the next section, we explore mechanisms that work against and could even ruin endosymbioses.

## Challenges of prokaryotic endosymbioses

In this section, we examine some of the issues of endosymbiosis specific to prokaryotes and the origin of mitochondria. We omit discussing issues related to phagocytosis.

### Symbiosis in syntrophic biofilms

Biofilms and microbial mats are commonly considered the cradles of multi-species metabolic syntrophies where microbial partners evolve complex dependence networks [25, 113]. According to syntrophic scenarios of the origin of mitochondria and eukaryotes, the merger between host and symbiont was initiated by a syntrophic interaction much like those in modern biofilms [17-19, 36-40], with the caveat that biofilms involve much more than two species and cheaters are bound to arise. The evolution and stabilization of dependencies in biofilms are proposed to be driven by two mechanisms, the Black Queen hypothesis [114] and the foraging-to-farming hypothesis [115].

The Black Queen hypothesis claims that a division of labor can evolve as a result of adaptive gene loss in microbial communities in which certain metabolites serve as ‘leaky’ public goods. Producers suffer a competitive drawback; hence, losing such functions has an advantage, given that others produce the diffusible good in the vicinity. The theory claims that the fastest evolving species wins by losing the function, and the last one retaining it carries the burden of producing for the entire community [115]. Such processes pave the way for stronger dependency between species, and in an ideal situation, the complete metabolic functionality will be distributed between community members, resulting in a metabolome [115]. Such division of labor can be stable, providing significant fitness advantage to the obligate symbionts compared to the metabolically autonomous wild type [116, 117]. Nevertheless, if partners differ in dependencies and in generation times, the one with a faster life-cycle can outcompete its partner, unless the slowest growing partner is indispensable for the association or has means to control its partner [49, 118].

Foraging-to-farming claims that obligate dependence evolves because of recurrent, or continuous, ecological interaction between partners complementing each other’s metabolic functioning [115]. Some initial asymmetry regarding the efficiency of producing a certain product can lead to the loss of functionality in the less efficient producer, as independency is not crucial any more [115]. Evolution can lead from loose, facultative associations with horizontally transferred partners, often called farming (externally), via tightened association and privatization of the symbiont and its product to obligate mutualism with vertical symbiont transfer (internal farming). The last step can be driven by genetic drift as the privatized and vertically transmitted symbiont’s population size becomes so small that accumulation of deleterious mutations becomes inevitable, as is expected by Muller’s ratchet [13].

### Reductive evolution and Muller’s ratchet

Obligate endosymbionts (especially parasites) undergo reductive evolution due to two factors. One is the loss of non-essential genes, especially if the host complements functionality [119]. The other is Muller’s ratchet [120], according to which a small, asexual population is subject to the accumulation of maladaptive or deleterious mutations resulting in gene and functionality loss [121, 122]. It is especially true for endosymbiont populations that are very small [123]. Gene loss degrades functionality in the symbiont rendering it dependent on the host, ultimately becoming incapable of free living. This inevitable spiral, called the ‘evolutionary rabbit hole’, leads to increased dependencies of the partners and have been reported in several cases of mutualisms (with eukaryotic hosts) [13, 48, 121, 123]. In nutritional symbiosis, in which during nutrient rich times, the selection pressure is relaxed for maintaining symbiont pathways, deleterious mutations may more likely degrade the symbioses [121]. As a result, obligate dependence evolves not necessarily because it was advantageous, but because it was inevitable given the bottleneck in symbiont population size, lack of recombination, and the accumulation of deleterious mutations [122]. The result can be robust partner fidelity feedback with aligned interests that emerges due to genetic drift and not due to the selection for better quality partners, as often hypothesized [13].

Extreme gene loss of symbiont can lead to a symbiont devoid of useful function which then becomes a burden for the host [121]. Reduction can be extreme (MROs [22]) and can ultimately lead to symbiont loss [14, 124] or replacement [125, 126]. On the other hand, once the endosymbiotic organelle has emerged, strong host-level selection can prevent the spiral [121].

Deleterious mutations occur in mitochondria and these do not often get fixed [121, 127, 128]. There is indication that the host can restrict the transfer of mitochondria with degraded functionality due to deleterious mutations in some cases [129], and thus, a sort of selective choice based on partner functionality has the potential to counteract the decaying spirals of mutualisms. Alternatively, recombination of short mtDNA sequences [130] and mitochondrial fusion (possibly originating from before LECA) could counter the accumulation of deleterious mutations and equilibrate nuclear-encoded proteins over all mitochondria in the host [131].

### Issues of syntrophic consortia

While syntrophy (in general, metabolic cross-feeding) seems to be widespread and the most common basis of symbiosis among prokaryotes, it is perplexing why no obligate, intimate symbiotic associations are known other than that of mitochondria. Syntrophy might seem to be a dead end that never leads to endosymbiosis. In turn, we discuss some issues that might account for the lackluster nature of syntrophy and that seriously undermine syntrophic theories of mitochondrial origins.

First, syntrophic communities are usually diverse, multi-species systems [25]. No case is known where strong pairwise obligate (endo)symbiosis emerged in such multi-species consortia; never was it modeled under what conditions pairwise interactions emerge in such a network.

Second, cooperative, complementary (labor-divided) microbial metabolic interaction networks might be unstable and prone to collapse due to cheaters. Theoretical models have demonstrated that exploiters (partial secretors with a reduced genotype) actually cheat on full secretors [132, 133]. It was found that the loss of a gene from a full secretor meant that the external product concentration (and thus overall group growth) is decreased, contrary to how the Black Queen hypothesis suggests the emergence of streamlined cooperative genotypes. For example, in synthetic communities, gene loss in secretors resulted in decreased external product concentration and lower overall group growth [134], rendering the efficiency of the Black Queen hypothesis questionable. Furthermore, specialized genotypes being able to modulate their secretion levels when others invest enough, can further destabilize cooperation [132]. If secretion rates are increased, it renders the collective more vulnerable to cheaters [133]. In in vivo experiments an initially complementary cooperation can easily collapse as one of the products is overdosed, while the other lags [135]. This leads to the vanishing of one product and either one or both species (depending on how dependent they were on each other). Consequently, the winning species is better off evolving the missing function for itself than to team up.

Third, stable cooperation often relies on partner choice/recognition—but are interactions specific in a diverse syntrophic biofilm? According to a fresh view of holobionts [41], taxonomic units can be and are indeed frequently replaced by other species with similar functionality, called phenotypic exchange. It is, therefore, more appropriate to view such interactions as inter-guild rather than pairwise [136]. Furthermore, as asexual reproduction and gene loss degrades the symbiont partner (see Reductive evolution and Muller’s ratchet), it is better for the host to replace it with a more efficient (perhaps genetically related) partner, as it happened to the proteobacterial endosymbionts of spittlebugs [125]. In a biofilm, this effectively means that functionally equivalent metabolic partners can freely be replaced within the consortium. Accordingly, these partnerships are not specialized and partners are not inherited strictly vertically. Consequently, the endosymbiotic unit emerging from such a scenario would show a more diverse genetic background with traces of ancient partnerships, assuming nuclear transfer of genes from, and residual adaptations to, ex-partners. All modern mitochondria go back to a single ancestor [137, 138] and we do not see genetically diverse MROs. The non-eukaryotic gene content of the eukaryotic genome correlates strongly with Alphaproteobacteria [139, 140] with no other significant bacterial contribution comparable in size to Alphaproteobacteria [141]. Either there were no competitors to mitochondria, or there were multiple closely related (potentially metabolically equivalent) Alphaproteobacterial partners before the establishment of the mitochondrial ancestor. However, the minor bacterial contributors to the eukaryotic genome might indicate (among others) that mitochondria have indeed emerged from an already diverse metabolic consortium (see [142, 143]). Due to the at least 1 billion years of streamlining ([144], but probably more [145]), it is very hard to find out whether (Alphaproteo)bacterial genes in eukaryotes originate from genetically closely related bacterial partners or diverged from a single ancestor. There may have been a long series of endosymbiotic partners all paving the road to successful mitochondria (cf [146].).

### A continuum between exploitation and mutualism

Recent experimental results support the theory that unilateral or mutual exploitation can also serve as the basis of endosymbiosis [58, 147]. Experiments with unicellular eukaryotes feeding accidentally on algae can result in highly dependent endosymbiosis in a resource-fluctuating environment [148]. It is not clear whether the alga receives any metabolic help in exchange of its photosynthate [149], though they can benefit indirectly from the partnership if this is their only chance to survive a harsh environment. This experiment is formally analogous to the bet-hedging strategy assumed by the farming hypothesis [53].

Somewhat contrarily to the above considerations, bacterial genes appearing in archaeal genomes could be due to occasional endoparasites leaving a footprint in the host’s genome [150]. They hypothesize that archaea were frequently parasitized and that the mitochondrial ancestor could have been just one of the invaders who managed to take a foothold after being trapped in the endomembranes of the host. It is certainly not unreasonable to assume a parasitic interaction at the origin of mitochondria (as was suggested previously by many, see list at [8]). Several mutually beneficial associations were suggested to have evolved from ancient parasitic infections [48, 50, 52] and there are artificially infected eukaryotic cells that after several years became dependent on their bacterial parasites [98, 151]. However, there is no direct support for a parasitic ancestral mitochondrion. Most importantly, there are no examples of bacteria preying on or parasitizing archaea [33]. Nevertheless, there are many cases of bacteria–bacteria endoparasitism (*Bdellovibrio bacteriovorus* [95, 152, 153], indicating the likeliness of bacteria to become parasites of other bacteria (and, of course, unicellular and multicellular eukaryotes).

In case of unilateral exploitation, on the other hand, control mechanisms enable the host to exert such a tight control over the symbiont that the interaction becomes disadvantageous for the latter. In an experiment, the ciliate host has practically enslaved the symbiont green alga striving to maximize the benefit-to-cost ratio by means of triggering a stress response in the symbiont [58, 154], resulting in a strict, exploitative control by the host over the symbiont and a strongly environmentally context-dependent interaction. In such cases, the retention of the autonomously-living form proves to be advantageous for the symbiont, which might hinder the evolution of a more dependent association [58, 59, 154].

### Controlling the symbiont population

Control mechanisms are not only important for selecting the most beneficial partner but also to avoid asynchronous reproduction of the partners. In some cases, cell-cycle synchronization of host and symbiont might happen before internalization: in *Chlorochromatium aggregatum* the host and its epibionts seem to divide synchronized and orchestrated [155, 156]; however, the mechanisms are unclear. After internalization of the partner, synchronization becomes even more important. Successive division of the host halves a slower growing population of symbionts, ultimately converging to zero density, called divisional dilution. This can be observed in ciliates with photosynthetic algae growing in the dark [157] (also in silico [53]). On the other hand, an internal parasitic population growing faster than the host will ultimately burst it. These render the interaction instable and temporal unless there are means to control the symbiont population. Presumably, the host’s symbiont uptake alone cannot counter the net deficit caused by elimination (of parasites), digestion (of farmed prey), extinction, and divisional dilution, and hence, autonomous symbiont growth within the host seems essential to compensate [53].

There are multiple mechanisms to control the internal population size. In case of metabolic coupling, host can regulate the partner’s reproduction by controlling nutrient feed. If the host is in control of partner uptake, it can simply cease capturing new individuals [48]. Synchronized cell division can be selected for and is evolutionarily stable if symbionts can limit their own cell division and if both parties’ benefit is large, as some theoretical results show [158]. Alternatively, if the symbiont is constantly growing faster, which can be seen as parasitism, host might evolve to counter symbiont by faster digestion, leading to a coevolutionary arms race [158]. While both are costly processes, controlled lysis (digestion) may return some benefit by salvaging the eliminated partner. While intracellular digestion is only known in eukaryotes, an (auto)phagocytic host to mitochondria is not ruled out yet [54, 104, 142, 159]. Modern mitochondria are subject to autophagy and this mechanism might have evolved in eukaryotes to control defective or corrupted symbionts, as was hypothesized by [160] based on the similarity of mitochondrial protein importing and eukaryotic autophagy‐related proteins.

## Conclusions

We have reviewed the ecology and evolutionary stability of inter-species microbial interactions along six orthogonal dimensions: dependence (facultative or obligate), physical proximity (symbiosis), net of costs and benefits (commensalism, mutualism, parasitism), type of benefit (nutrition, protection, transportation), type of investment (by-product, invested, purloined), and type of control (partner choice, vertical transmission, nuclear integration, etc.). Each actual (endo)symbiosis can be characterized as a point in this multi-dimensional space, where probably no single trait can be optimized independently of the others. Hence, any discussion of prokaryotic endosymbiosis must involve them all.

Obligate, irreversible endosymbiosis is the result of prolonged coevolution of host and symbiont. While mutualistic associations as well as symbioses are widespread among prokaryotes, endosymbiosis is restricted to the single (presumed) example of the ancestors of the eukaryotic cell and its mitochondria. The initial interaction that set off this partnership is debated, but a mutually beneficial relationship is often preferred, assuming such a relationship favors endosymbiosis by default. Consequently, there are many syntrophic theories of eukaryotic origin, assuming an inherently mutually beneficial interaction of ancestral partners. However, convincing theoretical or experimental results supporting the idea that syntrophy (or any directly mutually beneficial metabolic interaction) can actually lead to endosymbiosis are lacking. Many metabolically intertwined microbial assemblies exist—none of them seems to be in an advanced stage of endosymbiogenesis. Syntrophy (metabolic cross-feeding in general) is remarkably prevalent, and presumably was so ~ 2 billion years ago—how come that no other endosymbiosis emerged and survived till today involving only prokaryotes?

It is not enough for endosymbiosis that the interaction is mutually beneficial, it must also provide a selective advantage for the partnership over competitors (possibly other syntrophic partnerships in the neighborhood) and must account for partner control. Even if positive selection is granted, microbial syntrophy cannot explain how one partner gets inside the other. Protrusions or pockets of the host membrane might have been able to trap the symbiont (as was hypothesized based on the nature of the recently cultivated Asgard archaeon [101]; also earlier by [161]), but trapping is far from internalization. From a mechanistic point of view, the only known way that one cell can enter another is either phagocytosis (predation) or parasitism—none of them is mutually beneficial. Accordingly, partners might have asymmetrical relations and selection is not guaranteed to favor the “partnership” above individuals.

As a matter of fact, most of the symbiotic interactions in nature are not symmetric and are certainly not always beneficial for both parties. Likely, exploitation rather than mutualism is responsible for mutualistic symbioses among microbes [162, 163]. It is very unlikely that the ancestral host symbiont of eukaryotic origin, at the very onset of their joint history, met as a jolly joker pair with immediate mutual benefits, synchronized cycles, and negligible costs. Benefits are either direct or indirect, and can range from trophic to metabolic contribution, safe habitat providing or new habitat colonization, prudent provisioning, acquired resistance, etc. While certain types of benefits render mutualists better competitors, other effects enable the pair to escape direct competition to become better “explorers”. The latter option might have been crucial in mitochondrial origins, where the symbiont might have allowed the host to venture into oxic or colder environments or to grow bigger than its predators.

Costs also vary and could be considerable. At the extreme, autonomous reproduction is waived, to contribute more to the “greater good”. For the group to be favored by selection over independent individuals, group-level benefits must dominate over group-level costs—for individuals, costs could sometimes be enormous and benefits indirect. A partnership with reduced mean fitness at certain times can still enjoy selective advantage over individuals if the group can better exploit spatially or temporally heterogenous environments than individuals. If there is advantage for the group, partners benefit from staying and reproducing together. If mechanisms allow, this could lead to the inclusion of one party within the other.

However, having a symbiont internalized is not the end of story. Maintaining an internal population against dilution by successive fissions, degradation due to Muller’s ratchet and digestion by a possibly (auto)phagotrophic host are all issues that need to be tackled. Restocking symbionts from the outside to update the symbiont gene pool is one method to counter both divisional dilution and asexual genetic degradation. This suggests that the host might have a mechanism to actively capture prey (phagocytosis) or the prey was rather an invading parasite. If eukaryotic phagocytosis indeed stems from archaeal components [7], it is possible that it was evolved in an archaeal lineage that became the host.

The vast number of prokaryote–prokaryote mutualisms and the lack of standalone prokaryote–prokaryote endosymbioses suggest that endosymbiotic interactions are not easy to stabilize for a long time. This is certainly a result of the fast adaptation of prokaryotes to new conditions and the ease of which endosymbionts can streamline their genomes (cf [119, 164].) and can exchange partners (cf [41].). How could the mitochondrion stably remain in its host then? The obvious solution to this paradox would be the earlier (or contemporary) emergence of the cell nucleus, a safe place to protect host genome against hybridization with partner and parasite genes [150] and the place to integrate symbiont genes to exert central control and to mitigate the effect of Muller’s ratchet. Many hypotheses assumed a nucleated proto-eukaryotic host since [165], e.g., the Archezoa hypothesis [166]. While Archezoa were taxonomically disproved, their phylogenetic possibility was not ruled out yet. Similarly, a phagocytotic host to mitochondria remains a reasonable alternative to syntrophic or parasitic scenarios [54, 104, 142, 159, 167, 168], see Fig. 2. If the host was nucleated, it would mean that prokaryotic endosymbiosis is indeed extremely unlikely or transitional, and we should look for analogies of the endosymbiogenetic origin of eukaryotes among protists instead of among archaea and bacteria. However, whether mitochondria came after or before the nucleus and phagocytosis is not known yet.

While mitochondria and plastids seem to be the textbook cases of permanent, stable endosymbiosis, we must emphasize (as did [14]) that neither represent the end of their respective evolutionary histories. With the change of context (environmental or host conditions), functions and even endosymbiotic organelles can turn out to be hindrances that are ultimately lost. This has happened multiple times to plastid-bearing protists [169] and even to mitochondriate eukaryotes [124]. Whether the organelle could be reintroduced into these hosts by the artificial integration of a new bacterial partner remains to be seen in the lab. There are already cutting-edge experiments with genetically engineered symbionts: an auxotrophic, ANT-expressing *E. coli* was successfully integrated within a respiration-devoid yeast where the bacterium can export ATP while depending on host’s thiamine [170]. Similar tools can be employed to engineer endosymbioses with immediate purloined benefits for the host and dependence benefits for symbiont, due to auxotrophy. This line of research will become extremely important as for now any assumption on the endosymbiogenetic origins of eukaryotes (and in general, prokaryote–prokaryote endosymbiosis) is based on the single example of alphaproteobacterial integration into an archaeal or proto-eukaryotic host (of which the details are still debated).

## Data Availability

Not applicable.
